# *Streptococcus pneumoniae* carriage, antimicrobial resistance, and serotype distribution in children and adults from Paraguay in the post-vaccinal era

**DOI:** 10.3389/fpubh.2025.1584857

**Published:** 2025-05-26

**Authors:** Graciela Russomando, Norma Fariña, Selilah Amour, Lorena Grau, Rosa Guillen, Sonia Abente, Monserrat Aldama, Ingrid Hahn, Hector Castro, Mélina Messaoudi, Zunilda Sanchez, Valentina Picot, Florence Komurian-Pradel, Milen Milenkov

**Affiliations:** ^1^Instituto de Investigaciones en Ciencias de la Salud, National University of Asunción, San Lorenzo, Paraguay; ^2^Facultad Politécnica, National University of Asunción, San Lorenzo, Paraguay; ^3^Laboratorio San Roque, Asunción, Paraguay; ^4^Service d’Hygiène, Epidémiologie, Infectiovigilance et Prévention, Hospices Civils de Lyon, Lyon, France; ^5^Public Health, Epidemiology, and Evolutionary Ecology of Infectious Diseases (PHE3ID), Centre International de Recherche en Infectiologie (CIRI), Inserm U1111, CNRS UMR5308, ENS de Lyon, Université de Lyon 1, Lyon, France; ^6^Hospital Pediátrico “Niños de Acosta Ñu”, Ministerio de Salud Pública, San Lorenzo, Paraguay; ^7^Fondation Mérieux, Lyon, France

**Keywords:** *Streptococcus pneumoniae*, serotypes, vaccine, carriage, children, adults, Paraguay

## Abstract

**Introduction:**

Infections due to *Streptococcus pneumoniae*, including pneumonia and meningitis, are a leading cause of morbidity and mortality, especially in low-and lower middle-income countries (LMICs) worldwide. Most reviews highlight the geographical differences in serotype replacement and antibiotic resistance observed through invasive pneumococcal disease (IPD) surveillance, predominantly in high-income countries; however, data from many LMICs remain limited or poorly characterized. This study was conducted among healthy children aged 2–59 months and adults living in the same household, to determine pneumococcal carriage rates, serotype distribution, and the serotypes associated with antibiotic resistance profiles, following the introduction of PCV10/PCV13.

**Methods:**

Nasopharyngeal samples (NP) were obtained from 420 child/adult pairs between September 2018 and October 2019. Detection, serotyping, pneumococcal isolation and antibiotic susceptibility testing were performed using standardized protocols. Additionally, vaccine impact on serotype prevalence was assessed by comparison with a group of 100 healthy carriers under 5 years of age, recruited at the same hospital between 2010 and 2014, prior to vaccine introduction.

**Results:**

We observed higher pneumococcal carriage in children (39%) than in adults (20%) and limited intrafamilial transmission. Vaccine serotypes continue to circulate among children despite vaccination, accompanied by a rise in non-vaccine serotypes. Almost 11% of fully vaccinated children still carried vaccine serotypes. Antibiotic resistance to beta-lactams and macrolides has increased; nearly one-third of the isolates were multidrug resistant while multi-drug resistant pediatric isolates were predominantly associated with serotypes 19F and 19A.

**Conclusion:**

Our findings reveal worrying trends in the epidemiology of *S. pneumoniae* in Paraguay, including the persistence of vaccine-type serotypes among vaccinated children and an increasing resistance to antibiotics of isolated strains. Given the critical role of carriage studies in monitoring the impact of PCV in LMICs, the public health community should explore ways to improve their feasibility and cost-effectiveness and better integrate these efforts into routine vaccine preventable disease surveillance systems.

## 1 Introduction

*Streptococcus pneumoniae* (or pneumococcus) is a commensal of the normal oral microbiota of healthy humans but also a common pathogen, causing a variety of diseases, ranging from sinusitis and otitis media to life-threatening conditions such as pneumonia, meningitis, or sepsis. Children, the older adults and immuno-compromised people are the most at risk of developing invasive pneumococcal disease (1). Globally the pneumococcus is the leading cause of community acquired bacterial pneumonia, with more than 1 million deaths attributed annually, especially in low-and lower-middle-income countries (LMICs) (2).

Asymptomatic pneumococcal colonization of the nasopharynx is considered a key factor for developing invasive pneumococcal disease (3).

Most reviews highlight geographical differences in serotype replacement and antibiotic resistance in invasive pneumococcal disease (IPD) surveillance, predominantly in high-income countries; however, data from many (LMICs) particularly in Latin America and the Caribbean (LAC), remain limited and poorly characterized.

In recent years, treatment options for *S. pneumoniae* infections have been limited by increasing resistance rates to beta-lactams and macrolides, the most relevant antibiotic classes for dealing with pneumococcal infections which emphasized the need of effective vaccines (4, 5). Current pneumococcal vaccines are based on purified capsular polysaccharide antigens from the most frequent serotypes associated with invasive infections. Indeed, almost all invasive strains of *S. pneumoniae* express a complex polysaccharide capsule, considered as a major virulence factor for its protection role against host phagocytic and complement mechanisms (6). The capsular polysaccharide composition is highly variable among pneumococcal strains, with 100 structurally distinct capsular types (serotypes) described to date (7).

The first polysaccharide vaccine, licensed in 1977, targeted 14 major serotypes and was quickly followed by a second one, covering 23 serotypes (Pneumovax23), responsible for more than 85% of invasive pneumococcal infections worldwide (8). As this type of vaccines is poorly immunogenic in children, because of their inability to induce T cell immune response, multivalent glycoconjugate vaccines were developed, composed of pneumococcal capsular polysaccharide antigens, covalently attached to a diphtheria carrier protein (9). The first one, PCV7, targeting the seven most virulent serotypes, was introduced in 2000 and updated formulations have been released since, targeting 10 (PCV10), 13 (PCV13), and 20 (PCV20) serotypes or using different carrier proteins (10).

In Paraguay, *S. pneumoniae* is the main cause of pneumonia and meningitis of bacterial origin in children and adults (11). Regarding pneumococcal vaccines, PCV10 was added to the regular vaccination schedule in 2012, succeeded by PCV13 in 2017. Their impact on pneumococcal carriage in healthy individuals remains unclear, as to date, no reports on asymptomatic colonization in the post vaccinal era have been published in Paraguay.

In this study, we report the pneumococcal carriage rates among healthy children and adults with residence in Paraguay’s Central Department, including serotype distribution and antibiotic susceptibility profiles of the isolated strains.

## 2 Materials and methods

### 2.1 Study design and settings

The present work is an observational, descriptive study, conducted as part of a framework project for prospective multicenter research on pneumococcal colonization in children and adults within the GABRIEL network (Global Approach to Biological Research, Infectious Diseases and Epidemics in Low-income countries). Healthy adults and children under 5 years old, living in the same household and attending the “Niños de Acosta Ñu” General Pediatric Hospital (HGP), San Lorenzo, Paraguay were enrolled from September 2018 to October 2019 after signing an informed written consent. The “Niños de Acosta Ñu” Hospital provides medical care mainly to low-income families residing in the Central Department, which has a population of around 2 million (approximately 30% of the Paraguayan population). All adult/child pairs who (i) were suspected of a respiratory infection or of invasive pneumococcal disease (including meningitis and sepsis), or presenting any other clinical signs requiring hospitalization, (ii) unable to present the child vaccination card, (iii) were not living in the same household, (iv) denied signing the informed consent form, were excluded from the study. Epidemiological data included sex, age, number of people living in the same household, daycare or pre-school attendance (for children), other children under 5 years old in the household, PCV vaccination (for children), asthma, chronic illness other than asthma, smoking (for adults), recent antibiotic intake (in the last 3 months), and recent respiratory illnesses (in the last 3 months).

### 2.2 Sampling

Nasopharyngeal samples were collected using flocked nasopharyngeal swabs (FLOQswabs™, Copan Diagnostics, Murrieta, USA). Samples were stored at 4°C in STGG (skimmed milk-tryptone-glucose-glycerol) medium (12) and transported to the Instituto de Investigaciones en Ciencias de la Salud laboratory of molecular biology within 6 h.

### 2.3 Species and serotype identification by PCR

DNA was extracted using the QIAamp DNA Mini Kit (Qiagen, Hilden, Germany). Nucleic acids were extracted from 200 μL of each nasopharyngeal sample, containing 5 μL of a calibrated heat-inactivated suspension of *Streptococcus equi ssp. zooepidemicus.* Purified nucleic acids were eluted in 120 μL of water following the manufacturer’s instructions. Detection of *S. pneumoniae* was carried out using a duplex real-time PCR assay targeting the *lyt*A gene of *S. pneumonaie* and the *srt*C2 gene of *Streptococcus equi ssp. zooepidemicus*, the latter serving as an internal control (IC) for monitoring DNA extraction efficiency. Primers and probes sequences were: *lyt*A-forward primer: ACGAATAACCAACCAAACAAC, *lyt*A-reverse primer: CCAGTAGCCAGTGTCATTC, *lyt*A-TaqMan probe: 6-FAM-TCA**A**TC**G**TC**A**AG**C**CGTTCT-BHQ1, scrtC2-forward primer: GGAGCAAAGTCCTTTTGACG, *srt*C2-reverse primer: ACCCCCTTTTCAAGATGACC, *srt*C2-TaqMan probe: CAC**C**AA**T**AC**G**AA**T**CG**G**CTGT (letters in bold indicate LNA modifications). The *lyt*A assay is adapted from the method described by Carvalho et al. (13) with primer and probe sequences optimized for duplex compatibility with the *srt*C2 assay with a *lyt*A detection sensitivity of 1.1 genome copies per PCR reaction, equivalent to 220 genome copies per milliliter of the original sample. The specificity of both primers/probe sets was validated against a panel of 148 Streptococcus strains including 117 *S. pneumonoiae* strains (provided by the National Reference Center for *Streptococci*, University Hospital RWTH Aachen, Germany), one strain of *Streptococcus equi ssp equi*, one strain of *Streptococcus equi ssp zooepidemicus* and 29 strains of closely related *Streptococcus* species including *S. agalactiae, S. anginosus, S. australis, S. bovis, S. canis, S. constellatus ssp constellatus, S. constellatus ssp pharynges, S. cristatus, S. downei, S. ferus, S. gordonii, S. infantarius ssp coli, S. infantarius ssp infantarius, S. infantis, S. iniae, S. intermedius, S. mitis, S. oralis, S. mutans, S. oligofermentans, S. parasanguinis, S. peroris, S. pseudopneumoniae, S. pyogenes, S. ratti, S. salivarius ssp salivarius, S. sanguinis, S. sinensis, S. sobrinus* (provided by bioMérieux, Marcy l’Etoile, France), without any cross-reactions observed for either the *lyt*A or *srt*C2 assays. *LytA* positive samples were cultured on blood agar for 16 h at 37°C in 5% CO_2_ enriched atmosphere. One to eight colonies producing alpha-hemolysis were isolated and subcultured on blood agar with a disk containing 5 μg of optochin (Liofilchem, Roseto degli Abruzzi, Italy) placed on each plate. Susceptibility to optochin was interpreted following the manufacturer guidelines (14). For each sample, one to two optochin-susceptible colonies (inhibition zone >14 mm) were isolated and stored in STGG medium at 4°C for serotyping and antibiotic susceptibility analysis. Nucleic acids extraction from *S. pneumoniae* isolates was performed from a single colony, resuspended in 200 μL of phosphate-buffered saline, and eluted in 120 μL of water.

Serotyping of both nasopharyngeal samples and *S. pneumoniae* isolates was performed using a multiplex real-time PCR assay, targeting the 40 most prevalent *S. pneumoniae* serotypes (15).

The reaction mixture for both PCR assays contained 5 μL of template DNA, 1X Takyon No ROX probe Mastermix dTT (ref. UF-NPMT-C0701, Eurogentec, Seraing, Belgium) and 80–180 nM of each primer and probe (depending on the primers/probe pair) in a final volume of 18 μL. Reaction conditions for both PCR assays were as follows: 95°C for 5 min followed by 45 cycles of 95°C for 15 s and 60°C for 60 s. Samples generating a *lytA* amplification curve with a threshold >40 cycles were considered negative. Samples not generating the expected IC amplification curve were considered invalid. For those, DNA extraction and PCR testing were repeated. The evolution of serotype prevalence in children since vaccine introduction was analyzed, based on results from a study that we conducted in 2010–2014 (16).

### 2.4 Antibiotic susceptibility testing

Antimicrobial susceptibility testing of isolated strains was performed on a Vitek2 system (bioMérieux, Marcy-L’Etoile, France). AST-ST03 cards (bioMérieux, Marcy-L’Etoile, France), were inoculated as recommended by the manufacturer, using the Vitek DensiChek densitometer to calibrate the inoculum to a McFarland standard of 0.5–0.63 in 0.45% sodium chloride. *S. pneumoniae* ATCC 49619 strain was used as a control. The following antimicrobial agents were tested: penicillin, cefotaxime, ceftriaxone, levofloxacin, moxifloxacin, erythromycin, clindamycin, linezolid, teicoplanin, tetracycline, tigecycline, chloramphenicol, rifampicin and trimethoprim/sulfamethoxazole. Antimicrobial susceptibility results were interpreted following the Clinical and Laboratory Standard Institute (CLSI) guidelines (30th ed., January 2020). For penicillin, cefotaxime and ceftriaxone, meningeal and non-meningeal breakpoints were considered as CLSI considers useful in epidemiologic surveillance studies to report beta-lactam resistance by means of both meningitis and non-meningitis breakpoints.

### 2.5 Statistical analysis

Proportions of *S. pneumoniae* carriers and of serotype prevalence in the two groups were compared using the Chi-square test. Mean crossing threshold cycle (Ct) values were compared using the two-tailed Wilcoxon-Mann–Whitney test. To evaluate risk factors for *S. pneumoniae* carriage, a logistic regression was performed with *S. pneumoniae* carriage being the dependent variable. Variables with a *p* ≤ 0.1 in the univariate analysis were considered in the logistic regression analysis. A multivariate analysis was performed with adjustments on household size, smokers within the household, *S. pneumoniae* carriers among accompanying adult, age, gender, age of siblings living in the same household, daycare or pre-school attendance, PCV vaccination status, asthma and chronic illness other than asthma. The statistical analysis was performed using the Stata SE software v.17 to select the best model with minimum Akaike information criterion (AIC).

## 3 Results

### 3.1 Pneumococcal colonization

**Figure 1:**
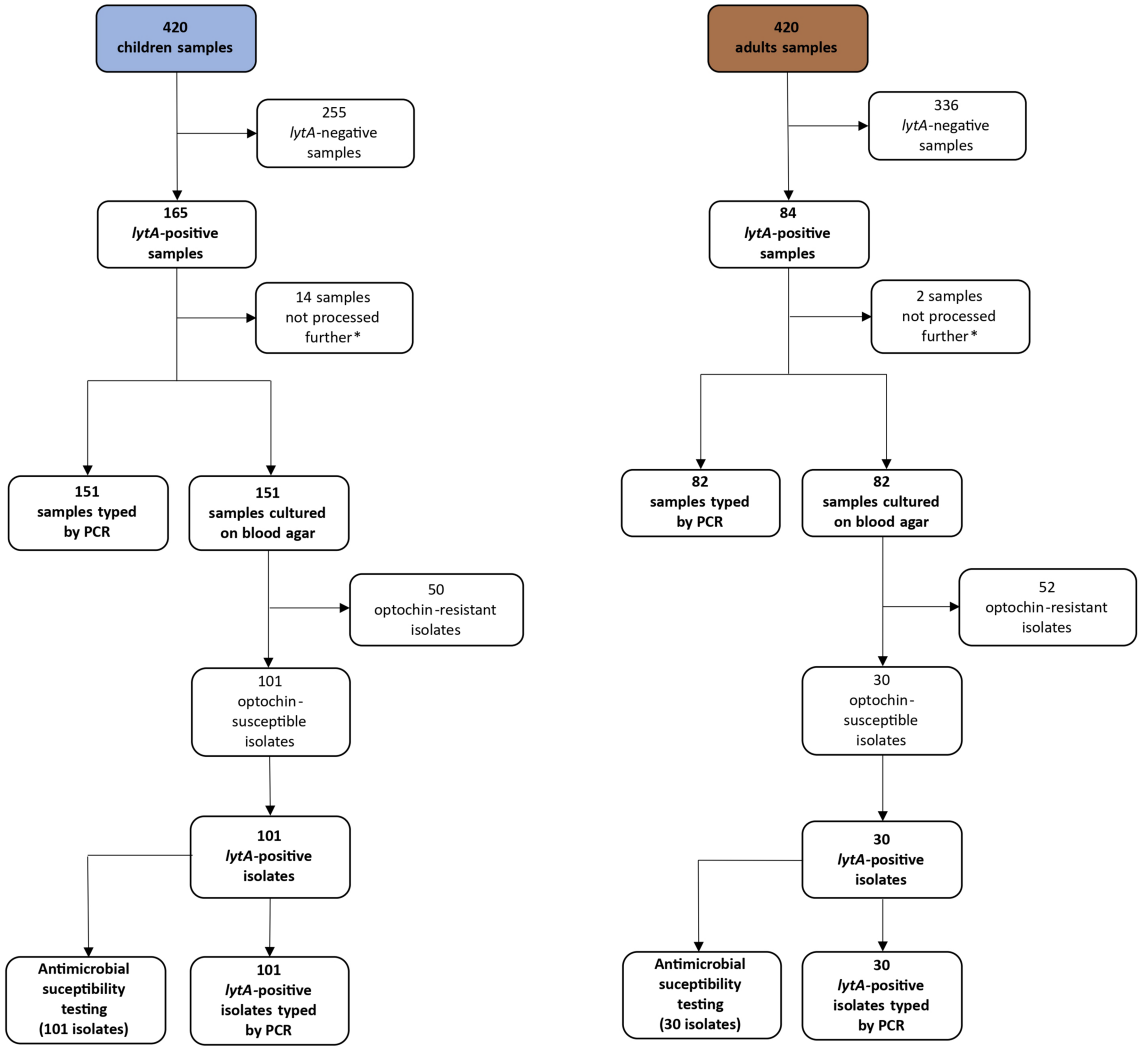
Flow chart representing the evolution of the number of samples throughout the study. *Culture, typing, and antimicrobial susceptibility testing were not performed for the last 16 samples due to reagents constraints.

Nasopharyngeal samples were obtained from 420 child/adult pairs between September 2018 and October 2019 (Figure 1). Among children, 97.9% (*n* = 411) had received the appropriate number of vaccine doses in respect to their age (Table 1).

**Table 1:** Characteristics of study participants during sampling at “Niños de Acosta Ñu” General Pediatric Hospital, September 2018 to October 2019.

Participants		Variable	*N* (%)
All (*n* = 840)	Household size	≤3	162 (39)
		4–8	228 (54)
		≥ 9	30 (7)
Children (*n* = 420)	Age (years)	≤1	123 (29)
		1–2	118 (28)
		2–3	69 (16)
		3–4	53 (13)
		4–5	57 (14)
	Gender	Male	223 (53)
		Female	197 (47)
	Other children under 5 years old in the household	Yes	148 (35)
		No	272 (65)
	Daycare attendance	Yes	31 (7)
		No	389 (93)
	PCV vaccination	Not vaccinated	9 (2)
		1 dose	42 (10)
		2 doses	101 (24)
		Complete vaccination	268 (64)
	Chronic illness other than asthma (missing = 3)	Yes	36 (9)
		No	381 (91)
	Asthma	Yes	21 (5)
		No	399 (95)
Adults (*n* = 420)	Age (years)	≤20	29 (7)
		20–25	104 (25)
		25–30	97 (23)
		30–35	86 (20)
		35–40	72 (17)
		>40	32 (8)
	Other children under 5 years old in the household	Yes	148 (35)
		No	272 (65)
	Gender	Male	21 (5)
		Female	399 (95)
	Smoking	Yes	108 (26)
		No	312 (74)

**Figure 2:**
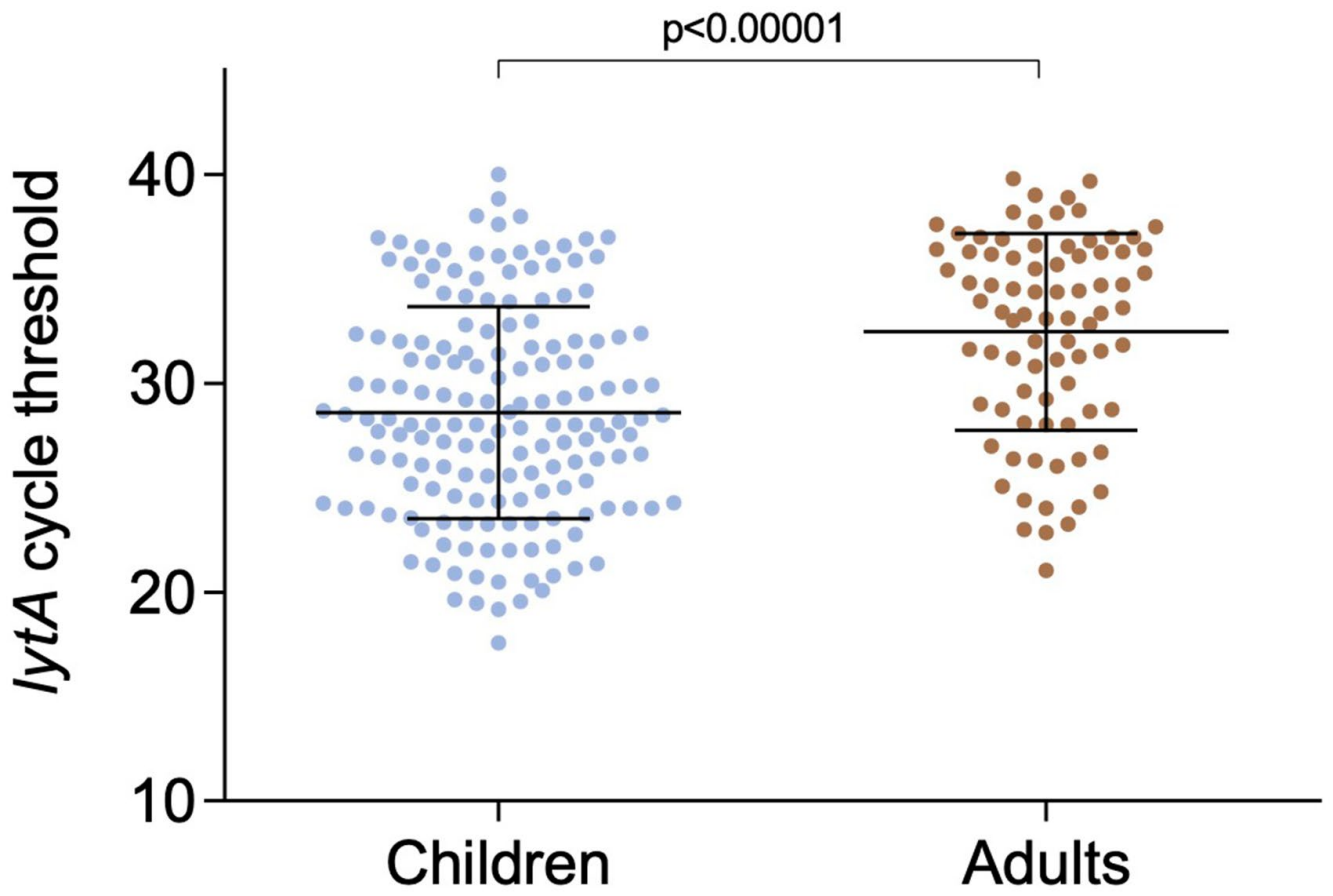
Real-time PCR cycle threshold values of *lytA* in children and adults nasopharyngeal samples.

The overall *S. pneumoniae* prevalence, based on *lytA* positivity rates of the nasopharyngeal samples, was 29.6% (249/840). Children were more frequently colonized (39.3%, 165/420) than adults (20%, 84/420) (*p* < 0.00001). Among the 420 child/adult pairs, 219 were lytA-negative, in another 119 only the child sample tested positive for *lytA*, and in 36 pairs only the adult sample was *lytA*-positive. For 46 pairs, both the child and adult samples tested positive for *lytA* by PCR. The mean *lytA* Ct value in the nasopharyngeal samples was 28.6 (range 17.6–40.0) in children and 32.5 (range 21.1–39.8) in adults (*p* < 0.00001) (Figure 2).

### 3.2 Risk factors for pneumococcal carriage

The only factors associated with pneumococcal carriage in children was pneumococcal colonization of the accompanying adult (OR = 2.34 95% CI 1.43–3.84, *p* < 0.01) and living with siblings under 5 years old (OR = 1.80, 95% CI 1.19–2.73, *p* < 0.01). In adults, the only risk factor for pneumococcal carriage was pneumococcal colonization of the child (OR = 2.37 95% CI 1.45–3.88, *p* < 0.01) (Table 2).

**Table 2:** Univariate and multivariate logistic regression of crude and adjusted risk for *S. pneumoniae* carriage in children and adults in Asuncion, Paraguay, September 2018 to October 2019.

Category	Variable		_crude_OR^†^	95% CI^α^	*P*-value	_adjusted_OR^‡^	95% CI^α^	*P-*value
Children^1^ (*n* = 420)	Household size	≤3	1 (Ref.)					
		4–8	1.06	0.70–1.60	0.782			
		≥ 9	1.66	0.76–3.62	0.207			
	smokers within household	No	1 (Ref.)					
		Yes	1.03	0.66–1.61	0.896			
	*S. pneumoniae* colonization of the accompanying adult	No	1 (Ref.)			1 (Ref.)		
		Yes	2.35	1.44–3.84	**0.001**	2.35	1.43–3.85	**0.001**
	Age (years)	≤1	1 (Ref.)					
		1–2	0.74	0.44–1.23	0.242			
		2–3	0.66	0.36–1.21	0.181			
		3–4	0.53	0.27–1.06	0.074			
		4–5	1.04	0.55–1.95	0.910			
	Gender	Male	1 (Ref.)		0.903			
		Female	1.02	0.69–1.52				
	Other children under 5 years old in the household	No	1 (Ref.)		**0.004**	1 (Ref.)		**0.005**
		Yes	1.82	1.21–2.74		1.80	1.19–2.73	
	Day-care attendance	No	1 (Ref.)					
		Yes	1.13	0.54–2.36	0.754			
	PCV vaccination	Not vaccinated or partially vaccinated	1 (Ref.)					
		Completed vaccinated	0.87	0.58–1.30	0.495			
	Chronic illness other than asthma (missing = 3)	No	1 (Ref.)					
		Yes	1.11	0.56–2.23	0.760			
	Asthma	No	1 (Ref.)					
		Yes	0.95	0.38–2.34	0.909			
Adults^2^ (*n* = 240)	Household size	≤3	1 (Ref)			1 (Ref)		
		4–8	0.74	0.45–1.23	0.251	0.73	0.44–1.22	0.225
		≥ 9	0.88	0.33–2.30	0.787	0.78	0.29–2.09	0.622
	Smokers within household	No	1 (Ref)					
		Yes	0.99	0.57–1.73	0.981			
	*S. pneumoniae* colonization of the child	No	1 (Ref)			1 (Ref)		
		Yes	2.35	1.44–3.84	**0.001**	2.37	1.45–3.88	**0.001**
	Other children under 5 years old in the household	No	1 (Ref)					
		Yes	1.22	0.17–2.01	0.424			
	Age (years)	Mean (±SD)	0.98	0.95–1.01	0.250			
	Gender	Male	1 (Ref)					
		Female	1.48	0.43–5.15	0.537			

_crude_OR^†^, Crude Odds Ratio; _adjusted_OR^‡^, adjusted Odds Ratio; 95% CI^α^, 95% confidence interval. ^1^The adjustment variables included in the final model were “*S. pneumoniae* colonization of the accompanying adult” and “Other children under 5 years old in the household.” ^2^The adjustment variables included in the final model were “Household size” and “*S. pneumoniae* colonization of the child”. Bolded values denote statistically significant *P*-values (< 0.05).

### 3.3 Serotype distribution in nasopharyngeal samples

**Figure 3:**
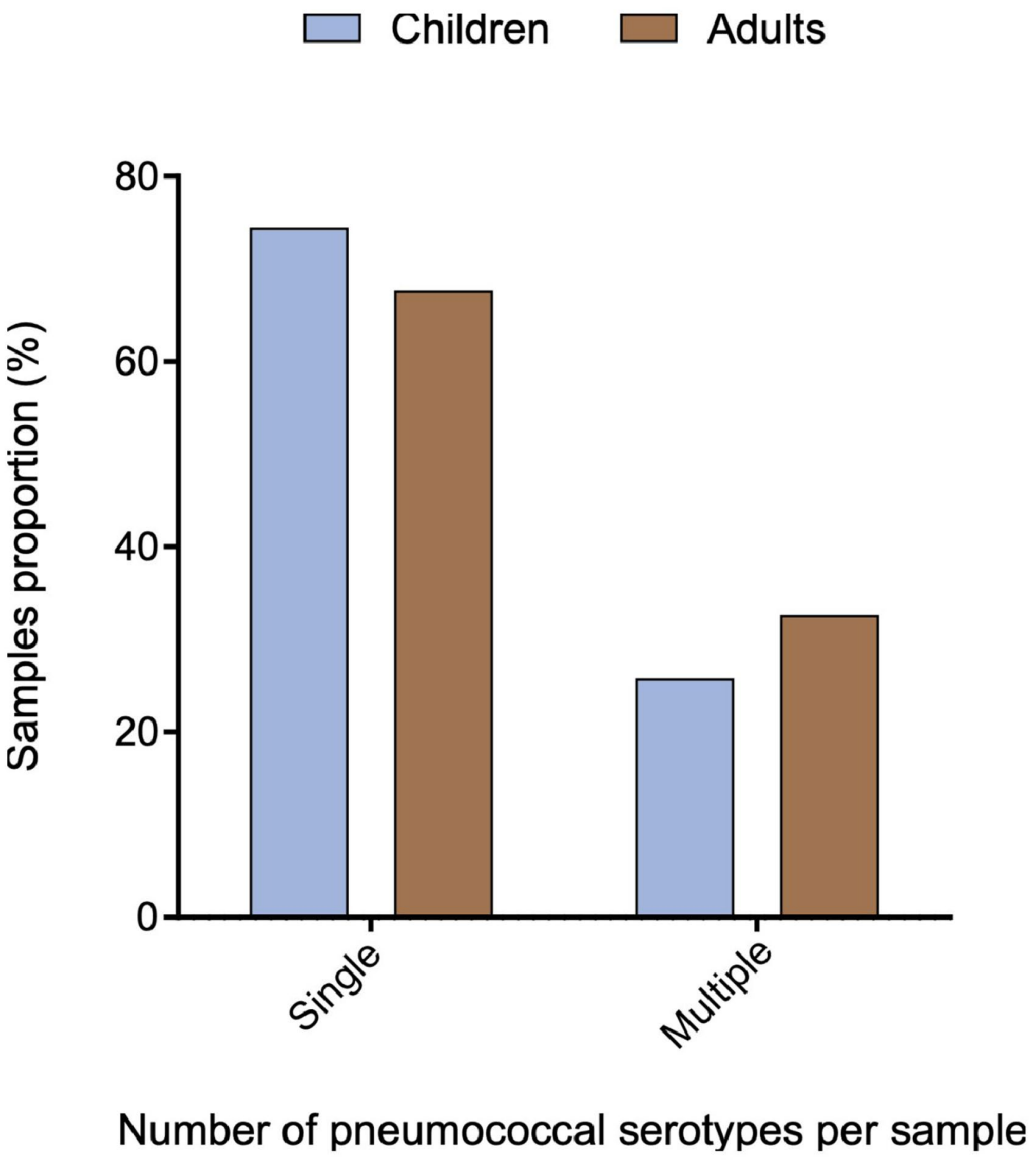
Proportion of nasopharyngeal samples containing a single or multiple serotypes among children and adults.

Serotype identification was performed by multiplex PCR on 233 of the 249 *lytA*-positive nasopharyngeal samples (151 children and 82 adults) (Figure 1). We identified at least one serotype in 65.2% (152/233) of the *lytA*-positive nasopharyngeal samples. No serotype was detected in 34.8% (*n* = 81, 42 children and 39 adults) of the samples (non typable samples). Single serotype carriage was detected in 71.7% (109/152) of the samples, whereas 28.3% (43/152) of the participants were colonized by two or more serotypes, without significant differences between children and adults (*p* = 0.31) (Figure 3). No statistically significant association was observed between single-or multiple-serotype carriage in children and factors such as vaccination status, age, gender or other socio-demographic variables including household size, smokers in the household, pneumococcal colonization in accompanying adults, siblings under 5 years old, day-care attendance or chronic illness including asthma (data not shown).

**Figure 4:**
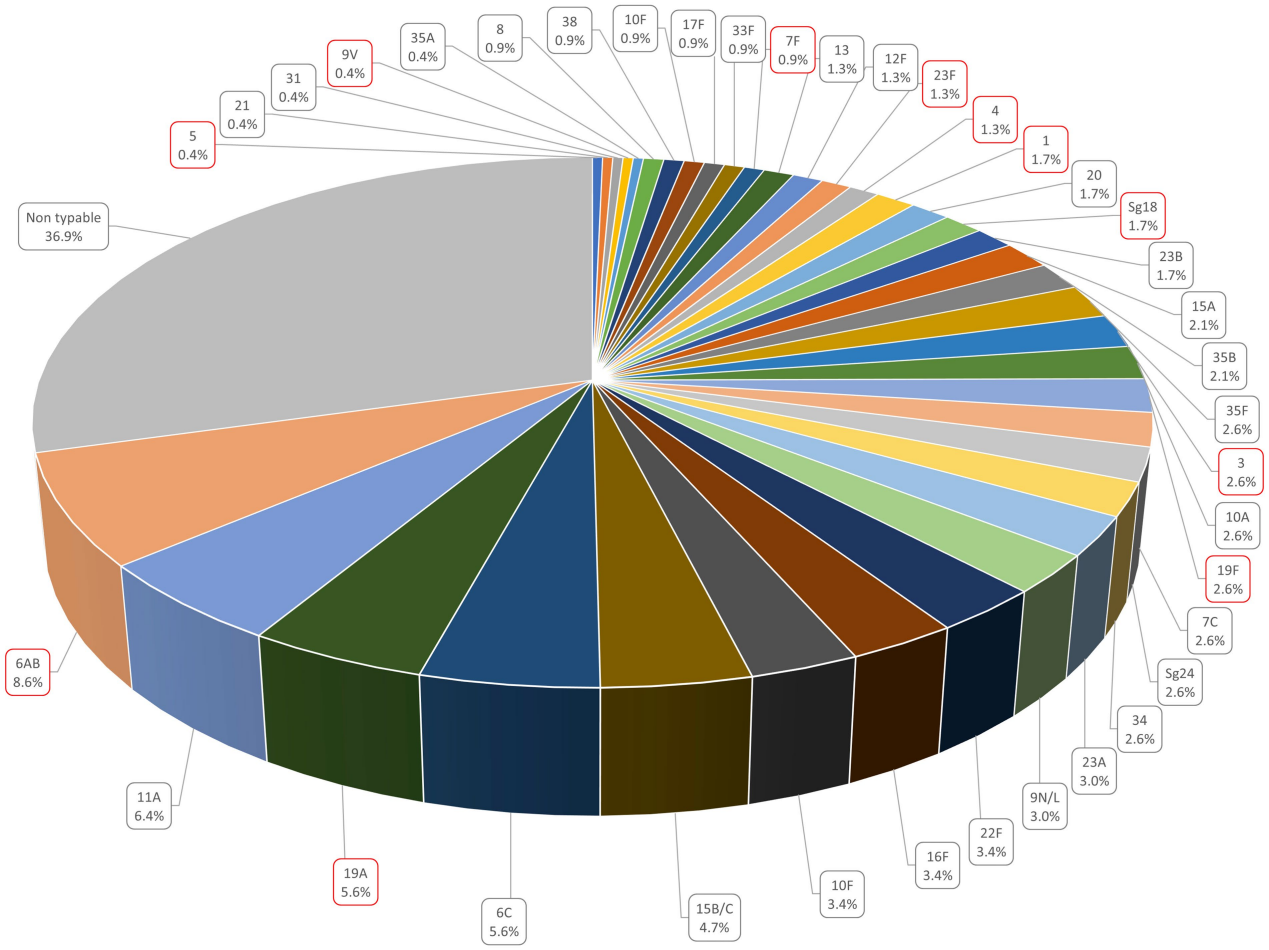
Serotype prevalence in the nasophary + ngeal samples. Serotypes surrounded in red represent those included in the PCV 13 vaccine.

**Figure 5:**
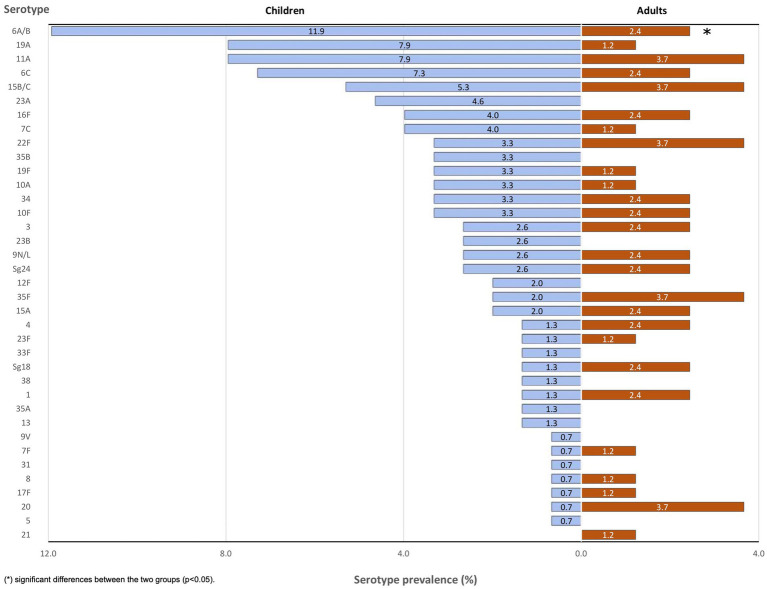
Serotype distribution in children and adults nasopharyngeal samples.

The most prevalent serotypes were 6A/B (8.6%, *n* = 20), 11A (6.4%, *n* = 15), 19A and 6C (5.6%, *n* = 13 each), and 15B/C (4.7%, *n* = 11) (Figure 4). Serotype prevalence between children and adults was comparable, with slight variations for most serotypes, except for 6A/B which was more frequent in children than in adults (*p* < 0.05) (Figure 5). In 17.4% (8/46) of *lytA* positive pairs, both the child and the adult were colonized by the same serotype.

### 3.4 Children vaccination status and serotype carriage

**Figure 6:**
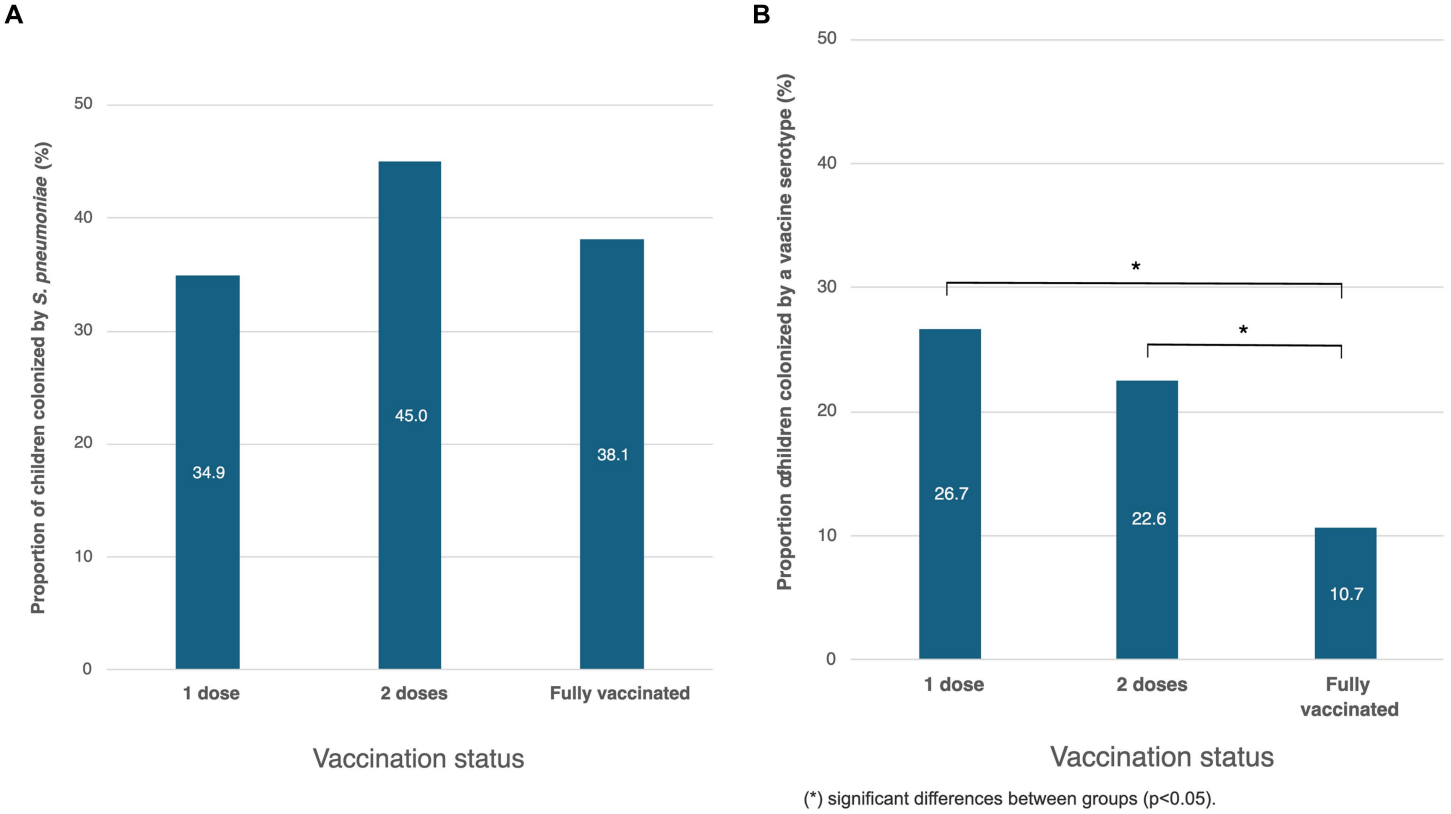
Association between children’s vaccination status and **(A)** *S. pneumoniae* carriage or **(B)** vaccine serotypes carriage.

Among the 420 children included in the study, 2.1% (*n* = 9) were not vaccinated, 10% (*n* = 42) had received a single dose of PCV10 or PCV13, 24% (*n* = 101) two doses, and 63.8% (*n* = 268) were fully vaccinated (Table 1). In all cases, partial vaccination was due to children’s insufficient age to be fully vaccinated. *S. pneumoniae* was detected in 41.4% (63/152) of non-vaccinated or partially vaccinated children and in 38.1% (102/268) of fully vaccinated ones (*p* = 0.69) (Figure 6A). Among children colonized by *S. pneumoniae*, a vaccine serotype was detected in 26.7% (4/15) and 22.6% (14/62) of those who had received one or two doses respectively, and in 10.7% (11/103) of fully vaccinated children (*p* < 0.05) (Figure 6B).

### 3.5 Vaccine impact on serotype prevalence

We also compared serotype prevalence in children to a group of 100 healthy carriers under 5 years old who were part of a study conducted at the “Niños de Acosta Ñu” General Pediatric Hospital between 2010 and 2014 (16). In this earlier study, l*ytA* was detected in 37% (37/100) of the participants. The most prevalent serotypes were 6A/B, 7C and 9 V, detected, respectively, in 18.9% (7/37), 13.5% (5/37) and 10.8% (4/37) of *lytA* positive samples. In 2018/2019, 6A/B remained the most prevalent serotype, although decreasing to 11.9% (18/151). In contrast, in our present study we found serotypes 7C and 9 V in only 4% (6/151) and 0.7% (1/151) of the children respectively, those being replaced by serotypes 11A and 19A (7.9%, 12/151 each). Of note, no 6C and 23A serotypes had been detected in 2010–2014, whereas they colonized, respectively, 7.3% (11/151) and 4.6% (7/151) of the children in 2018–2019. Concerning vaccine serotypes only, the prevalence of 9 V, 6A/B, Sg18, 1 and 7F decreased, serotypes 3 and 5 remained stable, while the prevalence of serotypes 4, 23F, 19A, and 19F increased. Except for serotype 9 V, these variations were not statistically significant. Globally, vaccine serotypes decreased by 31.9% (*p* = 0.78), whereas non-vaccine serotypes increased by 35.8% (*p* < 0.001) (Table 3).

**Table 3:** Serotype prevalence in children and evolution trends from 2010–2014 to 2018–2019.

Category	Serotype	N carriers		Prevalence (%)		Trend	Variation (%pt.)	Variation (%)	*p-*value
		2010–2014	2018–2019	2010–2014	2018–2019				
		(*n* = 37)	(*n* = 151)	(*n* = 37)	(*n* = 151)				
Vaccine-type (VT)	9 V	4	1	10.8	0.7	**↘**	−10.1	−93.9	**<0.001**
	6AB	7	18	18.9	11.9	**↘**	−7.0	−37.0	0.26
	Sg18	2	2	5.4	1.3	**↘**	−4.1	−75.5	0.12
	1	2	2	5.4	1.3	**↘**	−4.1	−75.5	0.12
	7F	1	1	2.7	0.7	**↘**	−2.0	−75.5	0.28
	3	1	4	2.7	2.6	**→**	−0.1	−2.0	0.98
	5	0	1	0.0	0.7	**→**	0.7	NA	0.62
	14	0	0	0.0	0.0	NA	0.0	NA	NA
	4	0	2	0.0	1.3	**↗**	1.3	NA	0.48
	23F	0	2	0.0	1.3	**↗**	1.3	NA	0.48
	19F	0	5	0.0	3.3	**↗**	3.3	NA	0.26
	19A	1	12	2.7	7.9	**↗**	5.2	194.0	0.26
	All VT	18	50	48.6	33.1	**↘**	−15.5	−31.9	0.78
Non-vaccine-type (NVT)	7C	5	6	13.5	4.0	**↘**	−9.5	−70.6	**<0.05**
	20	2	1	5.4	0.7	**↘**	−4.7	−87.7	**<0.05**
	17F	1	1	2.7	0.7	**↘**	−2.0	−75.5	0.28
	16F	2	6	5.4	4.0	**↘**	−1.4	−26.5	0.70
	38	1	2	2.7	1.3	**↘**	−1.4	−51.0	0.55
	12F	1	3	2.7	2.0	**→**	−0.7	−26.5	0.79
	15A	1	3	2.7	2.0	**→**	−0.7	−26.5	0.79
	35F	1	3	2.7	2.0	**→**	−0.7	−26.5	0.79
	15 BC	2	8	5.4	5.3	**→**	−0.1	−2.0	0.98
	10A	1	5	2.7	3.3	**→**	0.6	22.5	0.85
	31	0	1	0.0	0.7	**→**	0.7	NA	0.62
	8	0	1	0.0	0.7	**→**	0.7	NA	0.62
	13	0	2	0.0	1.3	**↗**	1.3	NA	0.48
	33F	0	2	0.0	1.3	**↗**	1.3	NA	0.48
	35A	0	2	0.0	1.3	**↗**	1.3	NA	0.48
	11A	2	12	5.4	7.9	**↗**	2.5	47.0	0.60
	9NL	0	4	0.0	2.6	**↗**	2.6	NA	0.32
	23B	0	4	0.0	2.6	**↗**	2.6	NA	0.32
	Sg24	0	4	0.0	2.6	**↗**	2.6	NA	0.32
	10F	0	5	0.0	3.3	**↗**	3.3	NA	0.26
	22F	0	5	0.0	3.3	**↗**	3.3	NA	0.26
	34	0	5	0.0	3.3	**↗**	3.3	NA	0.26
	35B	0	5	0.0	3.3	**↗**	3.3	NA	0.26
	23A	0	7	0.0	4.6	**↗**	4.6	NA	0.18
	6C	0	11	0.0	7.3	**↗**	7.3	NA	0.09
	NT	5	25	13.5	16.6	**↗**	3.0	22.5	0.65
	All NVT	24	133	64.9	88.1	**↗**	23.2	35.8	**<0.001**

### 3.6 Serotype diversity

Serotype diversity was higher in adults, with 26 different serotypes detected among the 82 adults (0.32 serotypes per participant), compared to 36 distinct serotypes among the 151 children (0.24 serotypes per participant), although this difference was not significant at the 0.05 level (*p* = 0.19). In contrast, serotype diversity in children in 2010–2014 (18 different serotypes detected among the 37 *lytA*-positive participants, 0.49 serotypes per participant) was significantly higher than in 2018/2019 (*p* < 0.01).

### 3.7 Pneumococcal isolation on blood agar

Nasopharyngeal samples positive for *lytA* by PCR, were cultured on blood agar, except for 14 children and 2 adults’ samples (Figure 1). Optochin-susceptible colonies grew on blood agar plates from 56.2% (131/233) of the nasopharyngeal samples. At least one pneumococcal strain was successfully isolated from 36.6% of adults’ samples (30/82) and from 66.9% of children samples (101/151) (*p* < 0.0001). PCR analysis of optochin-susceptible isolates confirmed the presence of the *lytA* gene in all isolates.

### 3.8 Antimicrobial susceptibility of isolated strains

**Figure 7:**
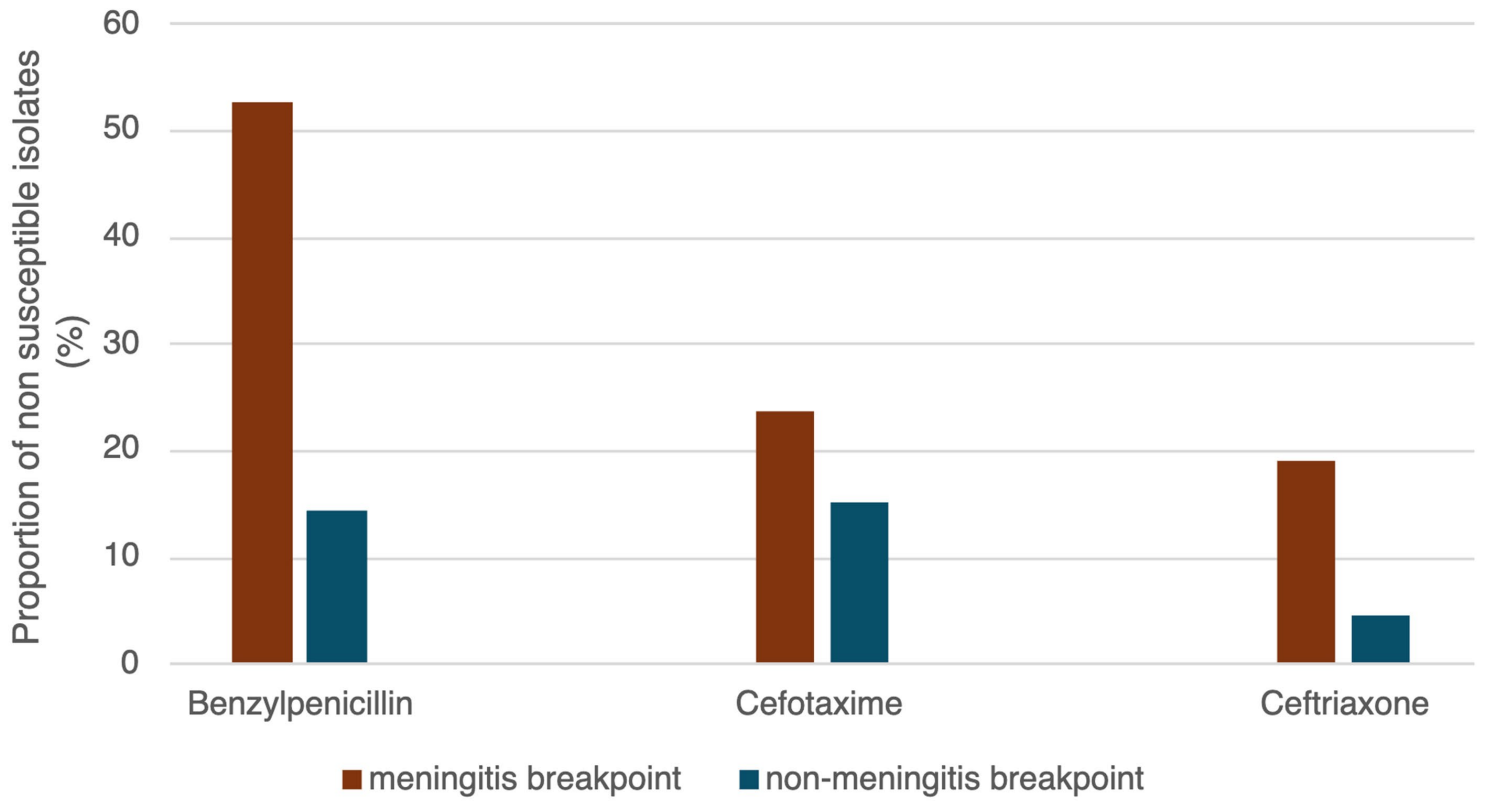
Proportion of isolates non-susceptible (resistant or intermediate) to beta-lactams considering meningitis and non-meningitis breakpoints.

Among the 131 isolates, 35.1% (*n* = 46) were susceptible to all antibiotics tested. We did not detect any resistance to linezolid, teicoplanin, tigecycline and rifampicin. Considering the meningitis breakpoints for benzylpenicillin, cefotaxime and ceftriaxone, 52.7% (*n* = 69), 23.7% (*n* = 31) and 19.1% (*n* = 25) were resistant or intermediate to these three antibiotics, respectively. Considering non meningitis breakpoints, resistance or intermediate susceptibility to benzylpenicillin was detected in 14.5% (*n* = 19) of the isolates, 15.3% (*n* = 20) to cefotaxime and 4.6% (*n* = 6) to ceftriaxone (Table 4 and Figure 7). Concerning other antibiotics, resistance was the highest for erythromycin and trimethoprim/sulfamethoxazole, 36.6% (*n* = 48) and 29.8% (*n* = 39) of the isolates being resistant or intermediate to these two antibiotics and in lesser extent to clindamycin (22.9%, *n* = 30), tetracycline (18.3%, *n* = 24) and chloramphenicol (3.1%, *n* = 4). A single isolate was resistant to levofloxacin and moxifloxacin (Table 4).

**Table 4:** Proportion of isolates resistant or intermediate to all antibiotics tested.

Antibiotic	Resistant *n* (%)	Intermediate *n* (%)
Benzilpenicillin (meningitis)	69 (52.7)	0 (0)
Benzilpenicillin (oral)	25 (19.1)	44 (33.6)
Benzilpenicillin (other)	4 (3.1)	15 (11.5)
Cefotaxime (meningitis)	21 (16)	10 (7.6)
Cefotaxime (other)	6 (4.6)	14 (10.7)
Ceftriaxone (meningitis)	5 (3.8)	20 (15.3)
Ceftriaxone (other)	3 (2.3)	3 (2.3)
Levofloxacin	1 (0.8)	0 (0)
Moxifloxacin	1 (0.8)	0 (0)
Erythromycin	48 (36.6)	0 (0)
Clindamycin	26 (19.8)	4 (3.1)
Linezolid	0 (0)	0 (0)
Teicoplanin	0 (0)	0 (0)
Tetracycline	24 (18.3)	0 (0)
Tigecycline	0 (0)	0 (0)
Chloramphenicol	4 (3.1)	0 (0)
Rifampicin	0 (0)	0 (0)
Sulfamethoxazole	26 (19.8)	13 (9.9)

For penicillin, cefotaxime and ceftriaxone, both meningitis and non-meningitis breakpoints were considered.

**Figure 8:**
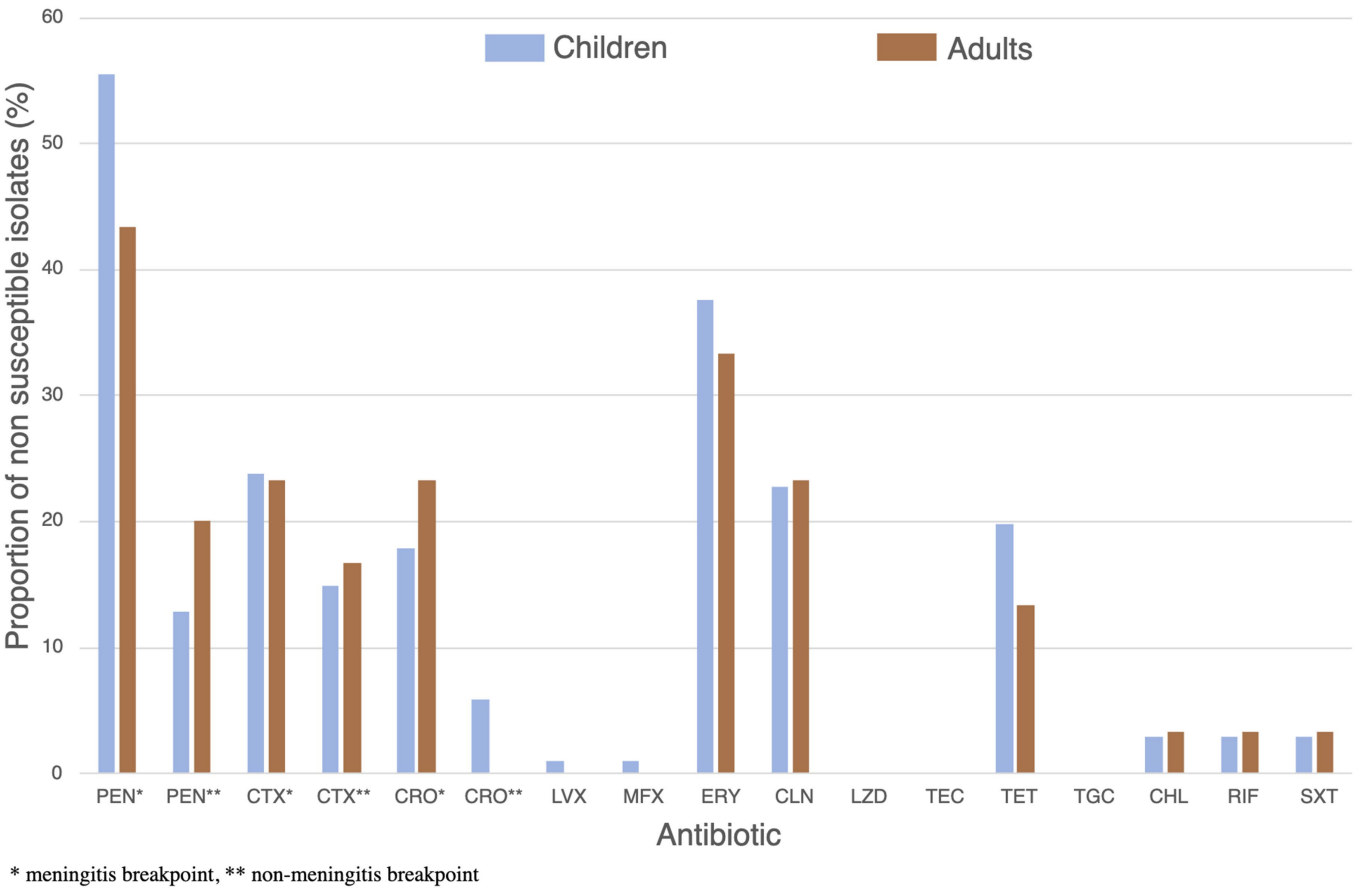
Proportion of children (blue) and adults’ (brown) isolates showing resistance or intermediate susceptibility to 14 different antibiotics. *Significant differences between group (*p* < 0.05).

**Figure 9:**
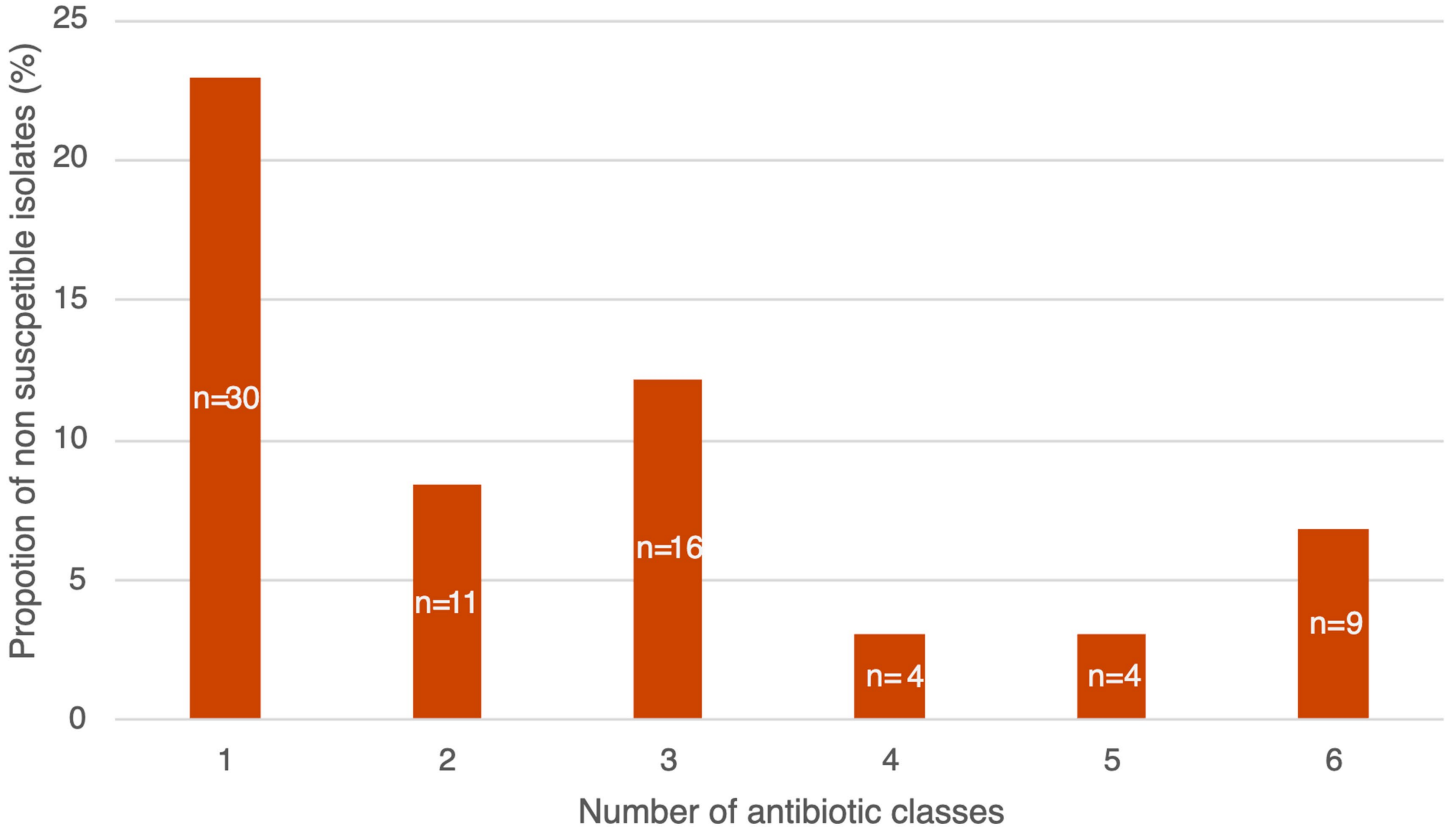
Proportion of non-susceptible (resistant and intermediate) isolates by number of antibiotic classes. For penicillin, cefotaxime and ceftriaxone non-meningitis breakpoints were considered.

**Figure 10:**
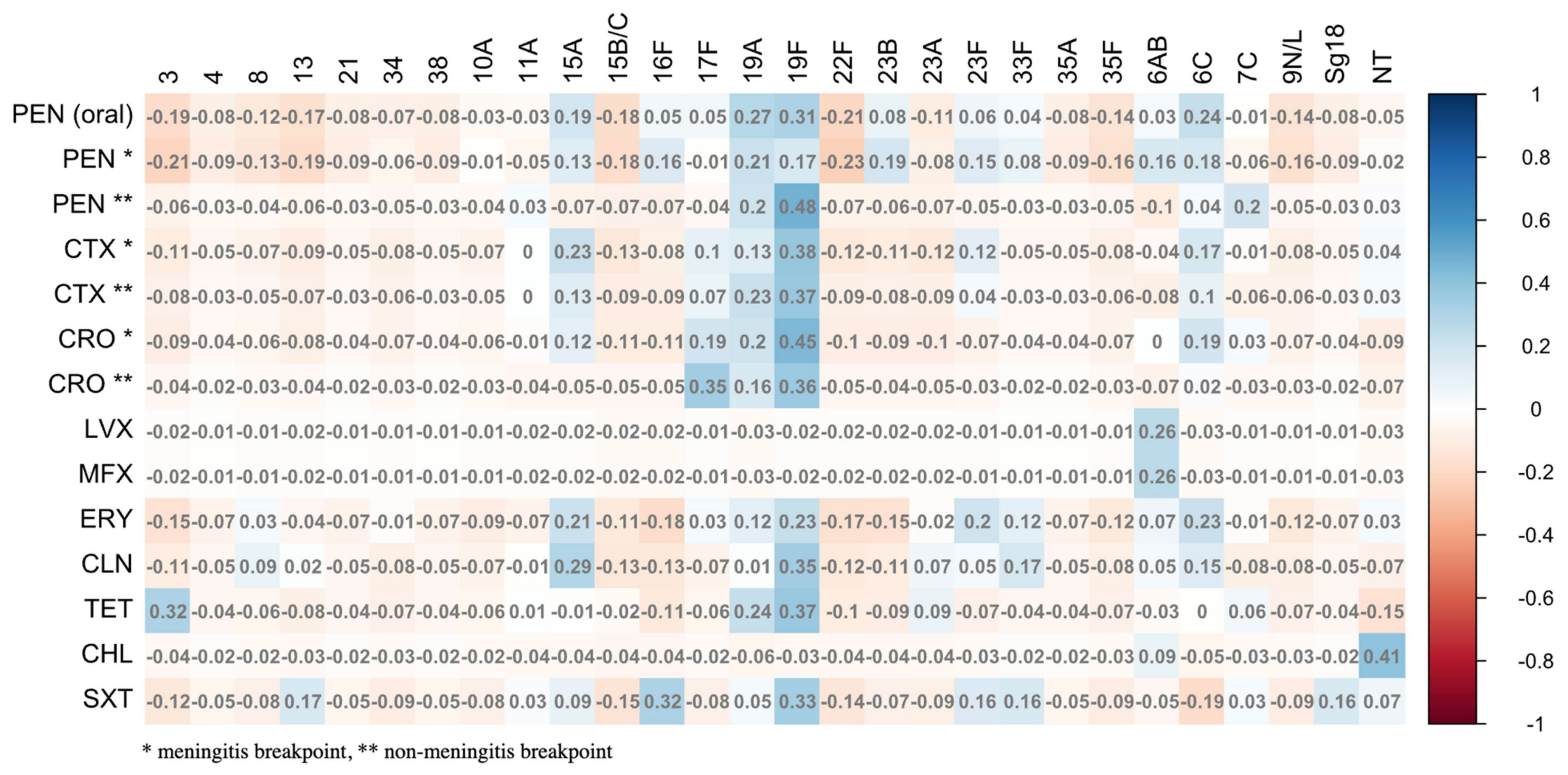
Relationship between serotype and antibiotic resistance profiles.

Globally, resistance rates were comparable between children and adults’ isolates without significant differences at the 0.05 level (Figure 8). Multidrug resistance was detected in 25.2% (*n* = 33) of the isolates, these being resistant or showing intermediate susceptibility to at least one antibiotic from three or more different classes (Figure 9). We did not observe a strong relationship between serotypes and antimicrobial resistance profiles, except for 19F isolates (*n* = 4) which had similar antimicrobial susceptibility patterns (Figure 10). Of note, all pediatric isolates (*n* = 8) resistant to 6 different antibiotic classes (benzylpenicillin, cefotaxime, ceftriaxone, erythromycin, clindamycin, tetracycline and trimethoprim/sulfamethoxazole) belonged to serotypes 19F and 19A (*n* = 4 each).

## 4 Discussion

A comprehensive understanding of *S. pneumoniae* colonization in healthy children is essential for assessing the impact of PCV vaccination on asymptomatic carriage rates and monitoring potential shifts in serotype distribution. Carriage studies also provide evidence of vaccine effectiveness against vaccine-type pneumococci in vaccinated individuals.

In the present study we not only evaluated pneumococcal colonization in children but also in their parents, which is important for evaluating the importance of intrafamilial transmission for *S. pneumoniae* dissemination in the community.

Adherence to the recommended vaccine schedule was high, with 98% of the children being vaccinated with the appropriate number of PCV doses in respect to their age. However, this does not necessarily imply uniform immunological protection across the entire cohort. Variability in dosing schedules such as delays in the administration of doses, missed booster doses, or incomplete primary series can influence both individual immune responses and nasopharyngeal colonization patterns. Children who have not received the booster dose, for example, may exhibit waning immunity, particularly against mucosal carriage of vaccine-type (VT) serotypes. Therefore, despite a high overall vaccination coverage, incomplete vaccination may introduce heterogeneity that should be considered when interpreting serotype carriage data (17).

In the context of Latin America, our findings add to a growing body of evidence reporting the persistence of vaccine-type (VT) pneumococci and the rise of non-vaccine-type (NVT) serotypes following PCV introduction (18). In our study, we observed an overall carriage rate of 29.6%, with a significantly higher prevalence in children (39.3%) compared to adults (20%). This pattern is consistent with studies from other countries (19–21), underlying the role of children as a primary reservoir of *S. pneumoniae*. Pneumococcal colonization in children was significantly associated with colonization of the accompanying adult and living with siblings under 5 years old, these risk factors having been reported in other Latin American studies (22, 23).

We also assessed child/adult pairs living in the same household to evaluate the extent of intrafamilial transmission. However, only 17.4% (8/46) of the pairs shared the same serotype. Several factors could explain this low serotype concordance: (i) adults may resist serotypes common in children (immune history); (ii) adults and children may acquire pneumococci from different sources (exposure differences); (iii) children carry more, and for longer, than adults (colonization dynamics). Adults may have developed serotype-specific immunity through repeated natural exposure events and previous infections, resulting in colonization by NVT or less immunogenic serotypes (3, 24).

Since our first study, conducted from 2010 to 2014 (16), pneumococcal carriage in children increased from 37 to 39%. Notably, in the present study, almost 11% of fully vaccinated children still carried at least one VT. These results suggest that not only PCV vaccination did not result in a reduction of the overall pneumococcal carriage in Paraguayan children, but also that VTs continue to circulate among Paraguayan children in the post-vaccinal era, especially 6A/B which remains the most prevalent serotype. This is consistent with reports from other low-and middle-income countries, where reductions in carriage of VTs after national-wide PCV vaccination campaigns have been modest (22–24).

As reported from other Latin American countries, we observed a decline of certain VT serotypes (1, 6A/B, 7F, 9 V, 18C) and an increase of a variety of NVT serotypes (6C, 9 N/L, 10F, 11A, 13, 22F, 23A, 23B, 24, 33F, 34, 35A/B). Overall, VT prevalence declined by 32%, while NVTs increased by 36% from 2010–2014 to 2018–2019. This serotype shift is in agreement with the global trends since PCVs introduction (25, 26). In particular, serotypes 6C and 23A are the most rapidly increasing NVTs in Paraguay, an observation reported from other countries since PCV introduction, vaccine serotypes being progressively replaced by non-vaccine ones (25, 27, 28). These changes are consistent with a recent Paraguayan study which revealed an increase in 6C and 19A and a decline in 6B among children with invasive pneumococcal disease (29). However, the global impact on invasive disease of this serotype shift in asymptomatic carriage is to be further investigated. In addition, serotype diversity was lower among vaccinated children compared to adults and to children of the pre-PCV period, likely reflecting the gradual replacement of VT strains by a limited set of emerging NVTs.

Regarding antibiotic susceptibility of isolated strains, we observed worrying trends concerning resistance to benzylpenicillin and ceftriaxone in comparison to a Paraguayan study conducted on healthy children under 5 years old in 2012, which did not report any resistance to benzylpenicillin and ceftriaxone considering non-meningitis breakpoints. Resistance to erythromycin and clindamycin increased from 18 and 10% to 37 and 20% respectively, whereas resistance to tetracycline, chloramphenicol and trimethoprim/sulfamethoxazole is comparable. Additionally, we found that nearly one third of the isolated strains exhibited multidrug resistance, and strains resistant to six different antibiotic classes represented more than 10% of all isolates with at least one detected resistance. These results suggest an overall increase in antibiotic resistance of pneumococcal strains in Paraguay, a tendency reported in many countries in recent years (4, 30–32). Interestingly, resistance rates were comparable between isolates from children and adults, suggesting a widespread community-level transmission of resistant strains. While higher resistance is often expected in pediatric populations due to more frequent antibiotic use (20), our findings suggest a broader circulation of resistant pneumococci and a spread of resistant strains independently of variable selective pressure. The high variability in AMR rates, both over time and across different epidemiological settings, indicates the need for local centers of laboratory excellence to provide data on local and regional antimicrobial susceptibilities over time following the introduction of relevant vaccines. While NP samples are convenient and easily accessible, their role in monitoring and guiding AMR for invasive disease is incompletely understood. Extrapolating the impact of PCV on AMR trends from one country or setting to another should be undertaken with caution, given the multitude of factors driving AMR, such as antibiotic prescribing practices and regulations of antibiotic use.

Our study has some limitations. First, we were unable to perform sampling of all members inside a household which could have brought additional insights about intrafamilial transmission. Second, our multiplex real-times PCR is unable to differentiate some closely related serotypes, such as 6A and 6B and detects only the 40 most prevalent *S. pneumoniae* serotypes. Therefore, we were not able to determine the serotype of 37% of the isolates. Despite these limitations, our study included a significant number of children and adults and brings important information about serotype prevalence in the post vaccinal era, intrafamilial transmission and antibiotic resistance rates of circulating strains.

## 5 Conclusion

Consistent with other studies, our findings show that vaccine serotypes are progressively being replaced by non-vaccine ones in Paraguay, following PCV introduction. The resistance rates observed were comparable in pediatric and adult isolates, suggesting a community-wide spread of resistant strains. The high levels of antibiotic resistance observed are particularly concerning, underscoring the need for sustained, regionally tailored surveillance of both serotype distribution and AMR in *S. pneumoniae*. Carriage studies remain a critical component for evaluating the impact of vaccination, particularly in LMICs, and must be strengthened by improving feasibility, reducing costs, and integrating them into routine surveillance of vaccine preventable diseases.

As the field moves toward broader PCV formulations, such as PCV15 and PCV20, ongoing monitoring is essential to inform vaccination strategies and mitigate the impact of antimicrobial resistance.

## Data Availability

The raw data supporting the conclusions of this article will be made available by the authors, without undue reservation.
